# Breaking malignant nuclei as a non-mitotic mechanism of taxol/paclitaxel

**DOI:** 10.46439/cancerbiology.2.031

**Published:** 2021

**Authors:** Elizabeth R. Smith, Xiang-Xi Xu

**Affiliations:** 1 Department of Obstetrics, Gynecology and Reproductive Science, University of Miami Miller School of Medicine, Miami, FL 33136, United States; 2 Department of Radiation Oncology, University of Miami Miller School of Medicine, Miami, FL 33136, United States; 3 Sylvester Comprehensive Cancer Center, University of Miami Miller School of Medicine, Miami, FL 33136, United States

**Keywords:** Taxol, Paclitaxel, Taxanes, Microtubules, Mitosis, Nuclear envelope, Cell cycle, Drug resistance, Drug mechanism

## Abstract

Discovered in a large-scale screening of natural plant chemicals, Taxol/paclitaxel and the taxane family of compounds are surprisingly successful anti-cancer drugs, used in treatment of the majority of solid tumors, and especially suitable for metastatic and recurrent cancer. Paclitaxel is often used in combination with platinum agents and is administrated in a dose dense regimen to treat recurrent cancer.

The enthusiasm and clinical development were prompted by the discovery that Taxol binds beta-tubulins specifically found within microtubules and stabilizes the filaments, and consequently inhibits mitosis. However, questions on how paclitaxel suppresses cancer persist, as other specific mitotic inhibitors are impressive in pre-clinical studies but fail to achieve significant clinical activity. Thus, additional mechanisms, such as promoting mitotic catastrophe and impacting non-mitotic targets, have been proposed and studied. A good understanding of how paclitaxel, and additional new microtubule stabilizing agents, kill cancer cells will advance the clinical application of these common chemotherapeutic agents.

A recent study provides a potential non-mitotic mechanism of paclitaxel action, that paclitaxel-induced rigid microtubules act to break malleable cancer nuclei into multiple micronuclei. Previous studies have established that cancer cells have a less sturdy, more pliable nuclear envelope due to the loss or reduction of lamin A/C proteins. Such changes in nuclear structure provide a selectivity for paclitaxel to break the nuclear membrane and kill cancer cells over non-neoplastic cells that have a sturdier nuclear envelope.

The formation of multiple micronuclei appears to be an important aspect of paclitaxel in the killing of cancer cells, either by a mitotic or non-mitotic mechanism. Additionally, by binding to microtubule, paclitaxel is readily sequestered and concentrated within cells.

This unique pharmacokinetic property allows the impact of paclitaxel on cells to persist for several days, even though the circulating drug level is much reduced following drug administration/infusion. The retention of paclitaxel within cells likely is another factor contributing to the efficacy of the drugs.

Overall, the new understanding of Taxol/paclitaxel killing mechanism—rigid microtubule-induced multiple micronucleation—will likely provide new strategies to overcome drug resistance and for rational drug combination.

## The Taxane Family of Chemotherapeutic Drugs

The class of taxane drugs, including paclitaxel (tradename-Taxol) and docetaxel (tradename-Taxotere), is among the most effective anticancer agents commonly used in clinics today to treat several major cancers, including metastatic breast, ovarian, prostate, lung, pancreatic, and cervical cancers [1–7]. Currently, a cisplatin (or carboplatin)/paclitaxel regimen following debulking surgery is a standard frontline chemotherapy for ovarian cancer [1,8–12], and a dose intensive regimen of paclitaxel is also used in salvage treatment following recurrence [10–12].

Despite the impressive clinical success of paclitaxel as a frontline and salvage cancer therapy [12–14], a major challenge is the development of drug resistance in recurrent cancer [15–19]. Extensive investigations led to the proposal of a list of possible mechanisms for the important clinical question of paclitaxel resistance [7,20]. However, the common ability of cancer cells to acquire taxane resistance indicates that another major mechanism(s) has not yet been uncovered [15,19,21].

Since investigation of new microtubule-stabilizing agents, such as epothilones (ixabepilone), laulimalide, and discodermolide, is under development [22–24], our understanding of the mechanism of taxanes and other microtubule-stabilizing drugs is important and may have a significant clinical implication in the years to come [5,25,26].

## Paclitaxel Binding to Beta-Tubulin within Microtubules and Their Stabilization

The discovery in the 1980s that paclitaxel binds and stabilizes microtubules [27–29] and inhibits mitosis [30,31] in culture cells propelled the development of the compound into a common anti-cancer drug [13]. Cell culture studies provided clear evidence that paclitaxel inhibited mitosis, and the mechanism that paclitaxel acts as a mitotic inhibitor quickly gained widespread acceptance and is now considered a dogma [7].

**Figure 1:**
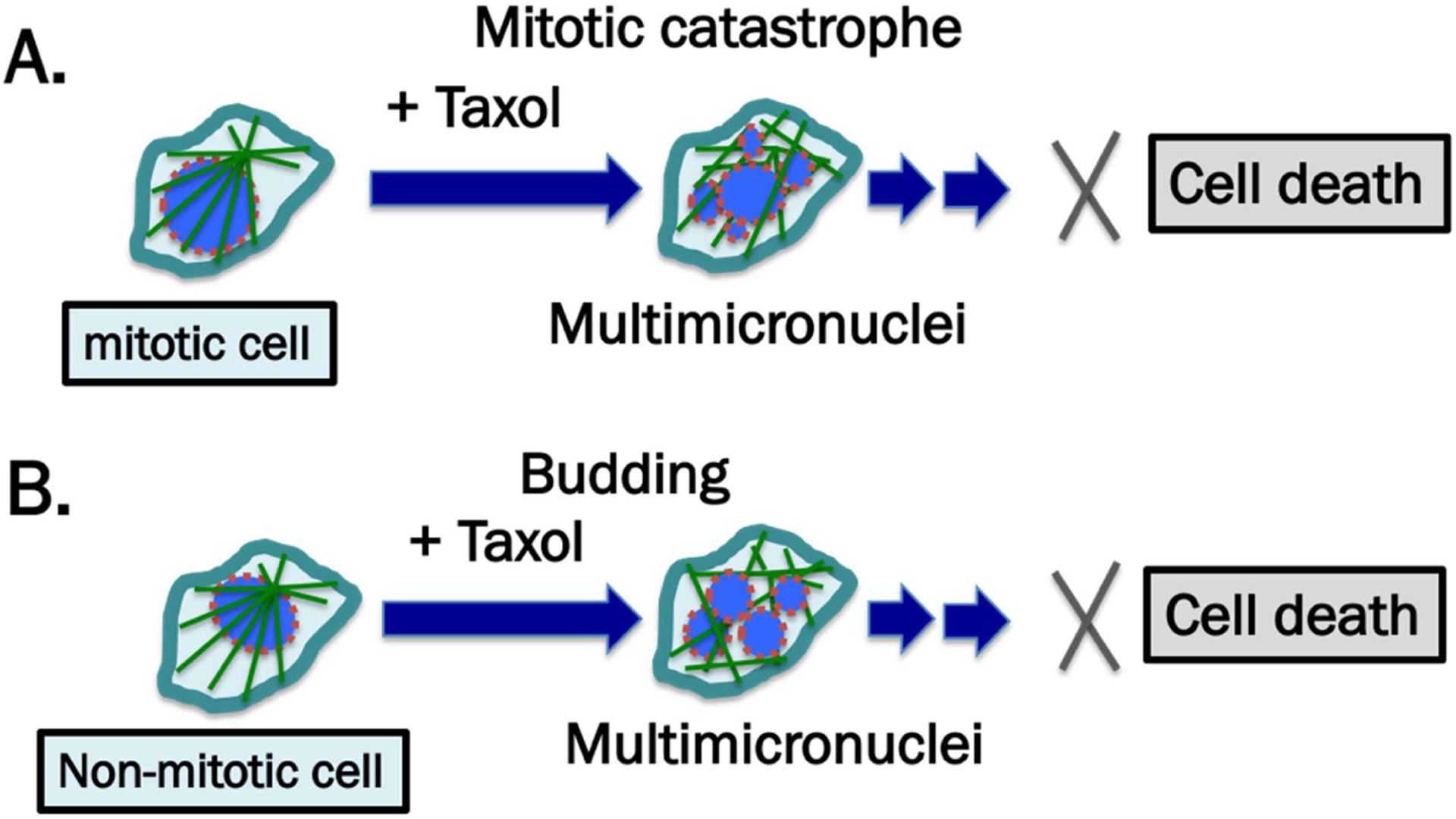
Mechanisms of paclitaxel in instigating cancer cell death by mitotic and non-mitotic mechanisms. Mitotic or non-mitotic cancer cells generally have weakened nuclear lamina, depicted by the broken brown-colored outlines of nuclear envelope. (**A**) The generally accepted mechanism is that paclitaxel binds microtubules and interferes with their function in chromosome segregation during the mitotic phase of the cell cycle. The cells escape mitotic arrest and undergo mitotic catastrophe and aberrant chromosome segregation and the resulting multi-nucleated and lobulated cells subsequently undergo cell death. (**B**) In addition to mitotic cell death, a new proposal is that in non-mitotic cells, the rigid microtubule filaments induced by paclitaxel can promote massive formation of micronuclei and nuclear multiple micronucleation by nuclear budding in cells during interphase. The multi-nucleated and lobulated cells die, through as yet not-well-defined mechanisms.

Generally, paclitaxel was thought to induce mitotic arrest and subsequently apoptosis in cancer cells [31–33] (Figure 1A). This idea seems reasonable and self-evident, as cancer cells exhibit uncontrolled growth and are usually more proliferative, and thus the targeting mitosis provides a specificity of paclitaxel for neoplastic compared to normal cells. Indeed, paclitaxel can cause significant off-target effects in normal, non-neoplastic cells that divide rapidly, such as hematopoietic cells [14] and cells of the hair follicle matrix [34], resulting in neutropenia and alopecia, respectively. Peripheral neuropathy is another dose limiting side effect of paclitaxel-induced microtubule stabilization [35].

Nevertheless, paclitaxel causes cancer cell death, rather than mere cytostatic [36–38], though how paclitaxel-induced cell growth arrest triggers death is not well understood [32,39,40]. However, binding and stabilization of microtubules is accepted as the key for the success of paclitaxel in cancer therapy [15,38]. Particularly, additional microtubule stabilizing molecules with chemical structures totally distinct from taxanes have been found to be effective anti-cancer agents [7,24,26,41]. Whether through mitotic or non-mitotic, apoptotic or non-apoptotic mechanisms, stabilization of microtubes has been found to be an amazingly optimal strategy in cancer therapy.

## Anti-Mitotic Mechanism and Mitotic Catastrophe

Further careful studies of the effects of paclitaxel on cancer cells in culture revealed that the cells often escape mitotic arrest and undergo aberrant mitosis [40,42]. Thus, an unsuccessful mitosis in the presence of paclitaxel-induced microtubule malfunction, a phenomenon known as mitotic catastrophe, may be a major mechanism of cell killing [43]. Experiments using time-lapse video microscopy revealed that paclitaxel-treated cells become multi-nucleated, often a result of multi-polar division [40,44–46]. An aberrant mitosis that forms multiple micronuclei, or nuclear lobules, as a result of paclitaxel arresting microtubules, is believed to be the major mechanism of drug action [44,47] (Figure 1A). The formation of micronuclei following paclitaxel treatment was initially observed many years ago [48,49], though it was only followed more recently. Generally, the formation of micronuclei is thought to be the result of chromosome mis-segregation during mitosis [45–47] (Figure 1A), although new observation suggest that paclitaxel also prompts the formation of multiple micronuclei in non-mitotic cells [50] (Figure 1B), as discussed below.

## Non-Mitotic Mechanisms and Prominent Formation of Multiple Micronuclei

One puzzle about the commonly accepted mechanism of paclitaxel action is the issue with mitosis as the target [51]. Unlike cells in tissue cultures, the neoplastic cells found in tumors in vivo are much less proliferative, with a doubling time significantly longer than cultured cells [42,52]. At any given time, only a small fraction of cancer cells are undergoing mitosis [37,38,42]. Thus, non-mitotic cells, in addition to cells undergoing mitosis, are likely targets of paclitaxel in cancer therapy [36]. Particularly in clinical settings, the susceptibility of cancer cells to killing by paclitaxel does not correlate with the proliferative index of the cancer [53]. This problem inspired the concept of “proliferative index paradox” [38], denoting that mitosis may not be a key target of paclitaxel or explain its efficacy as an anti-cancer agent [37,42,52,54].

Efforts to develop additional specific anti-mitotic agents inspired by the success of Taxol have not been successful [42,54,55], leading to skepticism about the rationale for targeting mitosis [37,52]. A few studies investigated and proposed non-mitotic mechanisms for paclitaxel in causing cancer cell cytotoxicity, including that paclitaxel influences bcl-2 phosphorylation [56]; paclitaxel targets microtubules involved in cellular transport [57]; and the drug impacts nuclear pores and transport [48]. Nevertheless, more investigations to identify a robust and general non-mitotic function of paclitaxel in targeting cancer cells seems warranted.

Indeed, recent studies showed that paclitaxel and other microtubule-stabilizing agents induce rigid microtubules that cause the breakage/fragmentation of the malleable nucleus of cancer cells, but not the sturdier nucleus present in normal cells [50] (Figure 1B). The paclitaxel-induced formation of multiple micronuclei is mitosis-independent, since paclitaxel-induced nuclear breakage still occurs when serum is removed to restrain growth, or in the presence of various mitotic inhibitors to suppress proliferation. Particularly, deletion of lmna gene (which encodes Lamin A/C proteins) sensitizes cells to nuclear breakage and death by paclitaxel [50]. Thus, a malleable nuclear envelope (caused by a reduction in Lamin A/C and perhaps other nuclear envelope structural proteins) underlies the specificity of microtubule stabilizing drugs such as paclitaxel in killing malignant cells.

The formation of multiple nuclear envelope fragments upon treatment of cancer cells with paclitaxel has been observed previously [48,49], though few studies have followed up the observation until recently. Generally, in the presence of paclitaxel to interfere with microtubule function, the formation of multiple micronuclei is thought to be a result of aberrant, multipolar mitosis [44–46].

**Figure 2:**
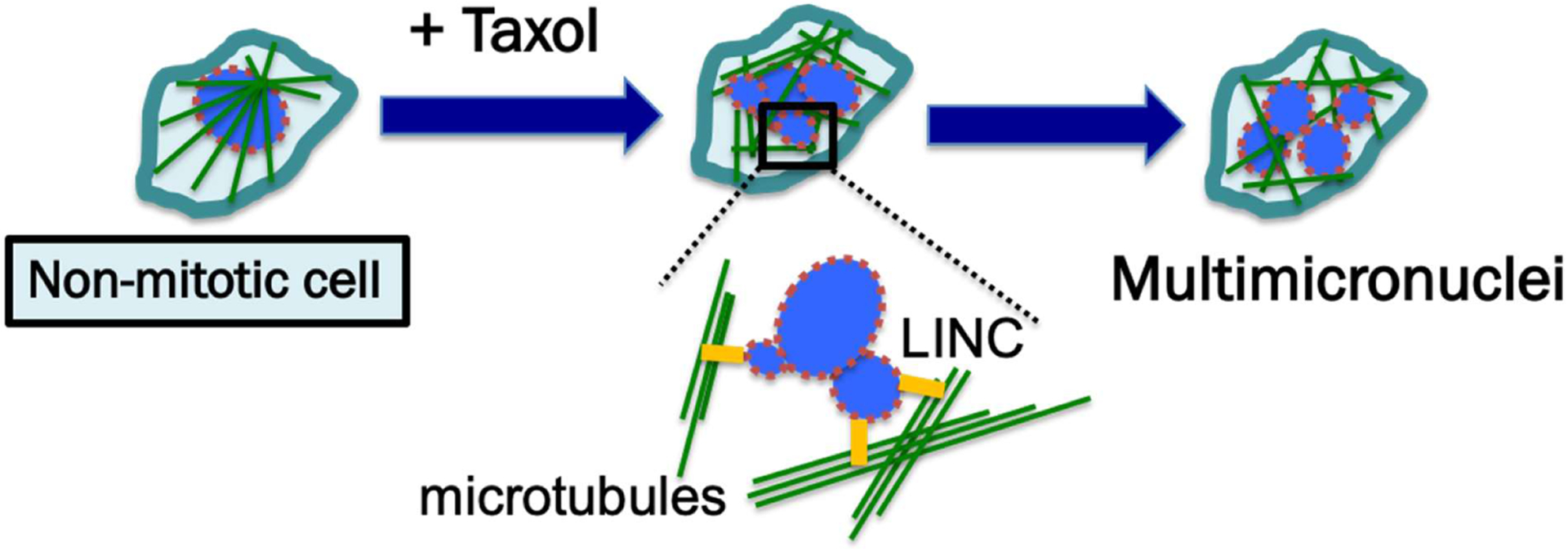
Proposed mechanism for the paclitaxel-induced formation of multiple micronuclei in non-mitotic cells. Cancer cells generally have weakened nuclear lamina, depicted by the broken brown outlines of nuclear envelope. A new proposal is that in nonmitotic cells, the rigid microtubule filaments induced by paclitaxel can promote massive formation of micronuclei through nuclear budding of cells during interphase. The paclitaxel-bound rigid microtubule bundles may physically pull and distort the nuclear envelope structure through the LINC (linking nucleus and cytoplasm) bridges, which connect microtubules and nuclear lamina. As a result, the malleable cancer nuclear envelope breaks into multiple micronuclei. The proposal of physical force exerted by paclitaxel-induced rigid microtubule filaments in breaking malleable cancer nuclei provides a non-mitotic mechanism to generate multiple micronuclei [50].

In the absence of drugs, nuclear budding occurring in non-mitotic cells may be an important mechanism in producing micronuclei [58–61], as microtubules associating with the nuclear envelope physically pull and distort the structure [62–64]. Similarly, the proposal of a physical force exerted by paclitaxel-induced rigid microtubule filaments in breaking malleable cancer nuclei provides a non-mitotic mechanism to generate multiple micronuclei [50] (Figure 2). The LINC (Linker of nucleoskeleton and cytoskeleton) bridges linking the microtubules to nuclear envelope lamina likely provide the physical links to transmit the force in pulling the nuclear envelope protrusion [62,63]. The proposed mechanism provides a possible alternative explanation for the well-established dogma that paclitaxel targets mitosis in cancer therapy; rather, paclitaxel likely aims at the weakened nuclear envelope of malignant cells. Thus, paclitaxel can be predicted to be effective to treat cancer that shows a deformed nuclear envelope, such as in the case of the cervical cancer cells that can be detected by a PAP test [65,66]. The study provides a new realization that paclitaxel can induce the generation of micronuclei in cells at S phase by a non-mitotic mechanism [50].

In addition, for paclitaxel to target proliferative, mitotic cells, the nuclear envelope malleability appears to be another characteristic of cancer versus benign cells targeted by paclitaxel. The loss or reduction of nuclear lamina proteins, especially Lamin A/C, in cancer cells has been previously noted [58–61]. Deletion of lmna gene encoding Lamin A/C is shown to lead to nuclear envelope malleability and paclitaxel-induced formation of micronuclei [50,67]. Thus, malleability of cancer nuclear envelope provides another specificity for paclitaxel.

## Cell Death Mechanisms Triggered by Paclitaxel and the Involvement of Micronucleation

The generally accepted concept is that in cancer chemotherapy, paclitaxel induces apoptosis [32,68]. This appears to be an intuitively reasonable idea, and there are many reports on induction of apoptosis in cancer cells by paclitaxel [39,69,70]. However, more careful studies indicate, at least in some circumstances, caspase activation and the typical or canonical apoptotic pathway are not involved [49,71–73]. Until now, how paclitaxel may trigger apoptosis is uncertain [15,68]. The lack of in-depth understanding of the cell killing mechanism of such a successful and common chemotherapy drug such as paclitaxel is surprising

**Figure 3:**
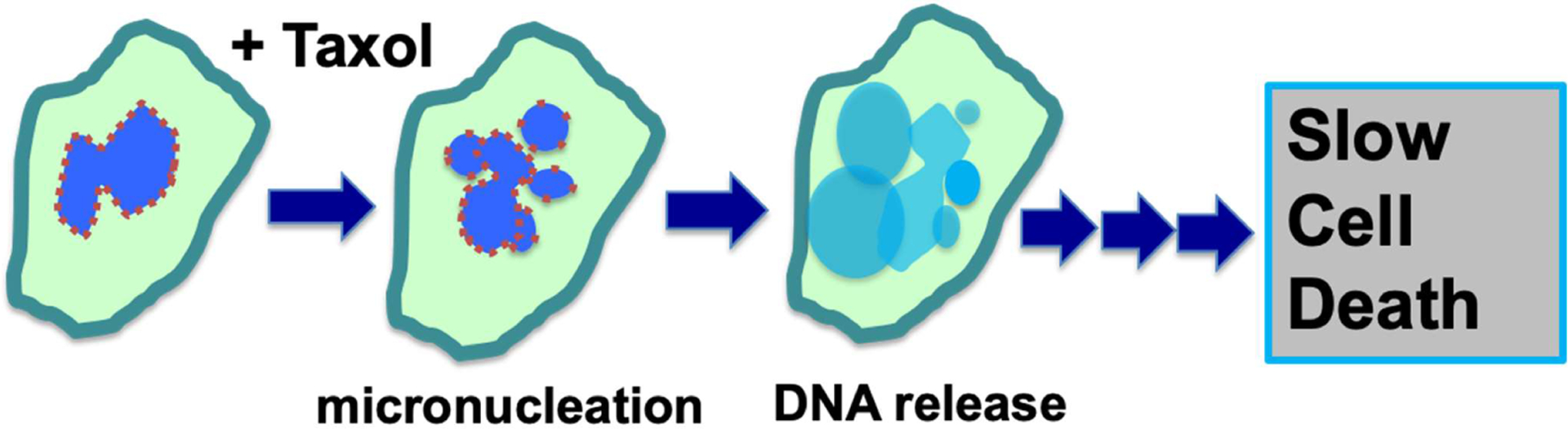
Mechanisms of paclitaxel-induced breaking of nuclear envelope and multiple micronucleation in cancer killing efficacy. Paclitaxel induces the breaking of nuclei of neoplastic cells and the formation of multiple micronuclei. The weaken nuclear envelope is depicted by the broken brown-colored outlines. The micronuclei are defective in membrane structure and have high propensity for rupture and release of chromatin material, resulting in compromised cellular structure and slow cell death.

Although the cancer killing mechanism of paclitaxel is not well understood, likely the formation of micronuclei induced by paclitaxel is important, referred as “micronucleation” [47,50] (Figure 3). How the formation of micronuclei leads to cell death is not established yet. In the absence of paclitaxel, micronuclei often undergo catastrophic rupture [58–60,74], which may lead to aneuploidy and cell death. Another notion is that the micronuclei formed may trigger innate cellular DNA sensing and subsequent induced immune pathways, which then contributes to cancer killing activity [47].

A suggested model is that paclitaxel eliminates cancer cells by first inducing “micronucleation”, the breaking of malleable cancer nuclei into multiple micronuclei (Figure 3). The membrane and lamina envelope of these micronuclei are defective, and are easily compromised structurally, resulting in the release of DNA content (Figure 3). Similar ideas have been suggested, that paclitaxel induced a slow, passive cell death without triggering apoptosis [71].

## Cellular Retention of Paclitaxel and Persistent Activity Within Cells

**Figure 4:**
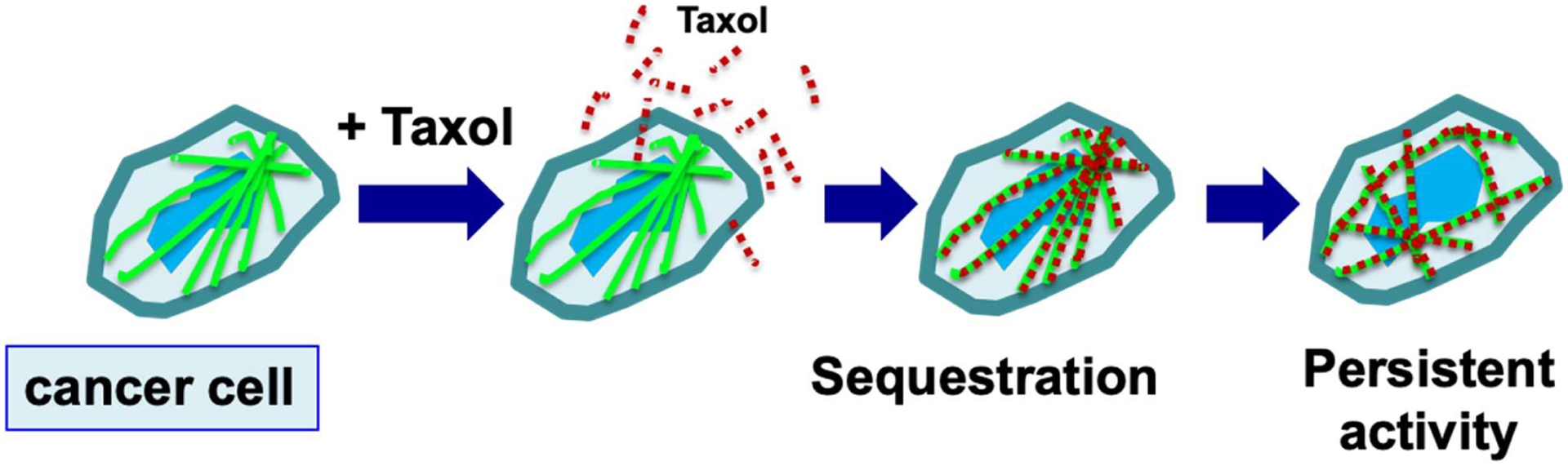
Retention of paclitaxel enables efficient killing of tumor cells. During chemotherapy, paclitaxel (Taxol) is administrated to patients over 3–6 hours, and taxane concentration reaches a peak level in plasma by the end of drug infusion. Over the next 6 hours, paclitaxel level declines rapidly, and the drug is concentrated in cells (partly by binding to microtubules) several hundred times over the blood level (illustrated by red dots). Paclitaxel is present in high level inside cells for next 2–3 days by binding to the microtubules, and the drug triggers nuclear envelope breakage and the death of cancer cells over the next 2–3 days, but also causes damage of hair follicles and toxicity in peripheral neurons.

Paclitaxel has high binding affinity to beta-tubulin located in microtubule filaments [27], and the binding can approach 1-to-1 ratio [75,76]. In a cell culture study, a short-term exposure of cancer cells to paclitaxel produces a long-term, persistent inhibition of cell proliferation and induction of cell death [77]. In vivo, although paclitaxel is rapidly cleared from the circulation following infusion, the drug is retained in cells and activity persists for several days [77–79] (Figure 4). Presumably, the high concentration of paclitaxel within cells interferes with microtubule-dependent cellular functions several days after drug administration. The retention of paclitaxel within cancer cells likely is important for killing of cancer cells, but the persistent presence of paclitaxel in peripheral neurons and hair follicles also causes the well-known side effects of paclitaxel, such as peripheral neuropathy [35] and alopecia [80].

Microtubules are polymers of alpha- and beta-tubulin heterodimers [76,81], and play multiple roles in cellular functions [81,82]. Cellular microtubule networks are highly dynamic: the filaments are constantly extending and shortening, with a balance between the cellular pool of alpha- and beta-tubulin dimers and microtubule polymers, which are about half and half under normal conditions [75,82,83]. Paclitaxel promotes 90–100% of tubulin monomers to locate into polymerized forms [76,82–84]. Because of the importance of microtubules in multiple cellular functions, the homeostasis and the level of free tubulins is tightly regulated [85–87]. Tubulins control their own synthesis by autoregulation at the level of mRNA stability [86,87]. Thus, addition of paclitaxel to eliminate alpha- and beta-tubulin dimers (into polymers) stimulates production of new tubulins. Production of new tubulins will further sequester paclitaxel, until all available paclitaxel molecules are eliminated.

Tubulins are relatively stable, and the tubulin protein is removed by proteasome- (but not lysosome-) mediated degradation [88] and via degradation by cathepsin D [89]. Cells take up, sequester, and concentrate paclitaxel at several hundreds of times over the concentration found in the extracellular space [75]. Indeed, intracellular paclitaxel can be retained over several days after exposure, during which time the paclitaxel bound rigid microtubules persist [75,77,79]. The ability of cells to uptake and concentrate paclitaxel results in part from paclitaxel sequestration by binding to abundant microtubules and tubulins (estimated to be in the range of 10–20 μM inside cells) [75,82,83].

Thus, a special feature of the pharmacokinetics of paclitaxel is the long retention of the drug inside cells from sequestration by binding to the ample cellular microtubules, despite rapid clearance of the molecules in circulation [75,77,79]. We speculate that the prolong retention is likely a factor contributing to the success of paclitaxel efficacy over non-microtubular targeting mitotic inhibitors and other anti-neoplastic cytotoxic agents (Figure 4).

## Prospects of the Microtubule Stabilizing Drugs with a Non-Mitotic Mechanism

Investigated in the 1970–1980s and entered into clinical use in the early 1990s [6,13,18], taxane/paclitaxel is still the most commonly used cancer drug today after treating millions of patients over the last 40+ years [13,23]. The development of taxanes for cancer therapy has been a celebrated success story [23,26], and new drugs with similar mechanism of actions as microtubule stabilization agents have a promising future in cancer treatment [24,41].

Paclitaxel is also used in combination with other agent(s), such as with doxorubicin (anthracycline) in metastatic breast cancer [2,3], and with Bevacizumab in lung cancer. The rationale combination of paclitaxel with other agents is an issue that oncologists must consider to increase the treatment response and efficacy [90,91]. Additional formulations such as Abraxane and liposomal taxane provide improvement on the delivery of paclitaxel and reduction of the hypersensitivity side effects [92,93]. Although the mechanism of paclitaxel drug action is still under study to gain a better understanding, microtubule stabilizing activity seems to be a key mechanism driving cancer killing activity [15,25]. A class of additional non-taxane microtubule-stabilizing agents, such as epothilones (ixabepilone), laulimalide, and discodermolide, isolated from microbiomes, sponges, and corals, respectively, is undergoing clinical development and testing in patient trials [22,24,41,94]. These new paclitaxel-like microtubule stabilizing agents may be useful for cancer that develops resistance to taxanes, and also for potential ability to be orally administrated, and have higher water solubility. Thus, continuing research and understanding of the microtubule stabilizing agents for their mechanism in efficient cancer cell killing will have a significant clinical implication in the years to come [13,23,26,91]. The newly uncovered non-mitotic mechanism of Taxol/paclitaxel in inducing breakage of cancer nuclear envelope [50]. (Figures 1–3) likely will prompt additional exploration and consideration in improving cancer chemotherapy using Taxol/paclitaxel and additional microtubule stabilizing agents.
